# Becoming Socialized into a New Professional Role: LPN to BN Student Nurses' Experiences with Legitimation

**DOI:** 10.1155/2012/946063

**Published:** 2012-03-29

**Authors:** Sherri Melrose, Jean Miller, Kathryn Gordon, Katherine J. Janzen

**Affiliations:** ^1^Centre for Nursing and Health Studies, Athabasca University, Athabasca, AB, Canada T9S 3A3; ^2^Mental Health and Addictions Services, Foothills Medical Centre, Calgary, AB, Canada T2N 2T9; ^3^School of Nursing, Faculty of Health and Community Studies, Mount Royal University, Calgary, AB, Canada T3E 6K6

## Abstract

This paper presents findings from a qualitative descriptive study that explored the professional socialization experiences of Licensed Practical Nurses (LPNs) who attended an online university to earn a Baccalaureate degree in nursing (BN), a prerequisite to writing the Canadian Registered Nurse (RN) qualifying exam. The project was framed from a constructivist worldview and Haas and Shaffir's theory of legitimation. Participants were 27 nurses in a Post-LPN to BN program who came from across Canada to complete required practicums. Data was collected from digital recordings of four focus groups held in different cities. Transcripts were analyzed for themes and confirmed with participants through member checking. Two overarching themes were identified and are presented to explain how these unique adult learners sought to legitimize their emerging identity as Registered Nurses (RNs). First, Post-LPN to BN students need little, if any, further legitimation to affirm their identities as “nurse.” Second, practicum interactions with instructors and new clinical experiences are key socializing agents.

## 1. Introduction

Vocationally educated Licensed Practical Nurses (LPNs) who enter an online university to upgrade their credentials by earning a Bachelor of Nursing (BN) degree can find the experience of socializing into a new and more complex professional role challenging [1, 2]. Professional socialization is the process of learning a professional role and emerging as a member of an occupational culture [3]. A key element within the overarching process of professional socialization is legitimation or the experience of gaining a sense of affirmation from socializing agents [4, 5].

Traditionally, opportunities for transitioning between vocational colleges and universities were limited [6–8]. Although few universities offer bridging programs for Licensed Practical Nurses, participants in the present study attended a new program where they were awarded prior academic credit for their previous nursing credential. Graduates of the bridging program go on to write the Canadian Registered Nurse (RN) qualifying exam. To date, educational research examining this group of nurses is limited.

This paper describes findings from a qualitative descriptive study that investigated the professional socialization experiences of 27 Post-LPN to BN students. Although most of the nurses' courses are offered online and are completed independently at their own pace, small groups of LPN to BN students do meet face to face for required practicum experiences. The practicum experiences are only offered in two Canadian cities, and students are required to travel to these locations for four-week periods. Over a period of two university terms, our project facilitators conducted focus group discussions with four different groups of students attending practicums. Post-LPN to BN students are required to complete one year of full-time experience as LPNs before they are admitted to the program. Participants in our focus groups were in their final cluster of required courses. By explaining students' perceptions of the legitimation experiences and socializing agents that helped them feel as though they were becoming Registered Nurses, we offer important insights for university educators who teach this new group of adult learners. Insights into the experiences that Post LPN to BN students themselves believe are affirming to their professional socialization can help educators facilitate clinical learning experiences that are relevant and meaningful.

## 2. Literature Review

### 2.1. Professional Socialization

Defining professional socialization is not straightforward. Socialization is a process where individuals acquire a personal identity and learn the values, norms, behaviors, and social skills appropriate to their social positions [9]. Professional socialization is a “process by which persons acquire the knowledge, skills and disposition that makes them more or less effective members (of a profession) … and a subconscious process whereby persons internalize behavioral norms and standards and form a sense of identify and commitment to a professional field” [10] (page 6). It includes the formation of an individual professional identity, where students come to view themselves as members of a profession with the knowledge and responsibilities which attend membership. It is thus an inherently social process [11].

In health care education, previous research has expanded our understanding of professional socialization. In medicine, the process includes both the intended and unintended consequences of an educational program [12], the informal implicit aspects of a “hidden curriculum” that can be more powerful than the “manifest” or official curriculum [13], and the preprogram attitudes that are important agents of socialization [14]. In social work, the process can include only limited changes in students' preprogram preferences [15] and the value and attitude dimensions have been identified as difficult to measure [16]. In physical therapy, the process is highly influenced by interactions with peers and faculty [17], by legitimation from socializing agents such as patients and clinical instructors [5], and by communication with practitioners [18].

In nursing education, previous research has examined professional socialization among select groups of student nurses, for example, traditional undergraduate nursing students [19, 20], undergraduate students specializing in community nursing [21], accelerated after degree students [22], male students [23], and students in distance programs [24]. Further, the experiences of select groups of Registered Nurses who upgrade their credentials have been explored. For example, upgrading to Nurse Anaesthetist [25]; to Nurse Practitioner [26, 27], and to Advanced Practice Nurse [28]. Finally, the legitimacy of nursing as an academic discipline has been examined [29].

Although an abundance of literature on professional socialization exists, there is a gap in our understanding of the experiences of vocationally educated nurses who attend university to earn their Registered Nurse (RN) credential. Kearney-Nunnery [30] explained that Licensed Practical Nurses are socialized to “collect client data and decide who needs to be informed,” while university educated Registered Nurses are socialized to “synthesize client data and make independent decisions” (page 19). Given the differences in role socialization between these two groups of nurses, when LPN to BN students undertake a mainly self-paced online curriculum, it is particularly important to examine the socializing agents that strengthen their feelings of legitimation when they meet faculty, peers, and patients face to face.

### 2.2. Legitimation

Legitimation, a critical element within the process of professional socialization, occurs when those around learners affirm that they are actually developing an identity as a member of their chosen profession [3]. Symbols, benchmarks, or “ritual ordeals” and people can all serve as valuable socializing agents during learners' experiences of legitimation [4].

As students are professionalized, they are initiated into a new culture wherein they gradually adopt those symbols which represent the profession and its generally accepted authority. These symbols (language, tools, clothing and demeanour) establish, identify and separate the bearer from the outsider, particularly from the client and the paraprofessional audience [4] (page 54).

In Haas and Shaffir's [4] view, early manipulation of these symbols of legitimization “heightens identification and commitment to the profession” (page 70) and more importantly “actually changes the neophytes' own perception of (self)” (page 72). Traditional symbols of legitimization in health care fields included white laboratory coats for medical students [13] and white caps for nursing students [3]. Today, name badges remain one of the few symbols of authority and legitimation that health care professionals continue to use as socializing agents. It is important to note that in the Post LPN to BN program, students' name badges do not include the identifier of “Registered Nurse.” Practitioners and patients who are not familiar with the program may not understand that Post LPN to BN students are experienced Licensed Practical Nurses developing new professional identities as Registered Nurses.

Additionally, benchmarks or “ritual ordeals” such as personal admission interviews, semester-based courses, and scheduled examinations for cohort groups also serve as socializing agents that bolster feelings of legitimation among learners in the health care fields [4]. Here again, students in the Post LPN to BN program do not participate in these benchmarking rituals. Their admission process did not include interviews, and they completed courses and examinations online at their own pace. Their only opportunity to meet faculty in person and join a cohort group was during their clinical practicums.

Finally, people such as faculty, peers, patients, and practitioners are important socializing agents that reinforce legitimation [4, 5, 17, 18]. Faculty evaluations of student progress and learning experiences that are new and different provide students with affirmation that they are progressing towards being granted professional legitimation and status. As Dall'Alba [31] emphasized “Learning to become a professional involves not only what we know and can do, but also who we are becoming” (page 34).

As part of an overarching program of research examining Post LPN to BN transitions, our research team questioned how Post LPN to BN students perceived their own processes of professional socialization and the kinds of formal and informal socializing agents of legitimation that contributed to or distracted from their growing identity as Registered Nurses.

## 3. Research Approach

This qualitative descriptive project was framed from a constructivist worldview [32–34] and Haas and Shaffir's [4] sociological theory of professionalization. Haas and Shaffir theorized that legitimation is a central concept in healthcare professionals' process of socialization. Participants were 27 Post LPN to BN students from a Canadian university who attended a practicum on an acute hospital unit. The main purpose of the research was to describe Post LPN to BN student nurses' experiences with professional socialization as they transitioned into a more complex nursing role. A secondary purpose of the research was to begin to understand how university faculty can best support and facilitate these students' professional socialization as they learn to become Registered Nurses (RNs). Data sources included four face-to-face digitally recorded, transcribed focus group discussions which were analyzed for themes.

Our rational for collecting and analyzing focus group data centered on our intention to invite our participants to converse and interact in ways that stimulated new insights. Focus group methodology, with its emphasis on group interaction [35–39] and goal of collaborative discussion [40, 41], allowed us to draw out participants' views and to explore their ideas and conversational exchanges with one another in depth. Focus groups are a rich source of information [42] and a valid method of generating data within a constructionist epistemology where “knowledge is created in situated, [collective] encounters” [43] (page 496). They are a useful method for gaining insight into phenomena where little is known [44]. When focus group data has been collected from multiple groups and multiple sites, researchers can have increased confidence in the reliability and validity of the findings [45].

The focus groups were guided by following questions.

1. Perceptions of Professional Socialization.
  - *What comes to mind when you hear the phrase “professional socialization”?*
  - *Share memories of your experiences becoming socialized into the role of Licensed Practical Nurse and developing your identity in this role.*
  - *How is the experience of developing your new role and identity as a Registered Nurse the same? How is it different?*
2. Formal Academic Experiences: Online Classes and Clinical Practicums.
  - *Talk* *about* *experiences you have had so far in your online university classes where you “felt like” a Registered Nurse and not a Licensed Practical Nurse? Have there been times in your online university classes where you haven't been sure about what it “feels like” to be a Registered Nurse? *
  - *Talk* *about* *experiences you have had so far in your practicums where you “felt like” a Registered Nurse and not a Licensed Practical Nurse? Have there been times in your practicums where you haven't been sure about what it “feels like” to be a Registered Nurse? *
3. Informal experiences (Employer Requirements, Workplace Interactions, and Existing Professional LPN Commitments).
  - *What have employers and colleagues at your workplace said or done that contributed to your “feeling like” a Registered Nurse? What distracted?*
  - *How do your existing professional Licensed Practical Nurse commitments contribute to your process of becoming socialized into the role of Registered Nurse? How do they distract?*
  - *Talk about the sorts of things that are going on in your life with family and friends that impact your changing role and professional identity.*

Transcripts from the focus group discussions were analysed for themes [46–48]. Our research team thoroughly read and reread the transcripts and met regularly to develop a systematic process of thematic analysis. We used investigator triangulation [49, 50] to create and agree upon the categorizations and coding schemes that led to our themes. Our themes appeared consistently in each of the four focus groups. Trustworthiness was established by member checking with participants to ensure authenticity.

Several strategies were utilized to increase rigor [45, 51]. Stability was enhanced through the use of multiple focus groups in geographically different areas. Equivalence was achieved through the use of two experienced moderators with complementary styles to achieve “flow, texture and context” and to promote construct validity [51] (page 302). Credibility was strengthened through sustained engagement and observation over the course of four focus groups, researcher triangulation, debriefing as a research team, and member checking. Reflexivity, where researchers strive to understand their own experiences as well as the research question, in order to remain objective, neutral, and nonbiased, was supported through regular face-to-face and teleconference meetings. Transferability was enriched through dense sample description and rich description of the data. Confirmability was heightened through peer debriefing and maintaining our audit trail. Dependability was attained by recording a log of our plans, meetings, and ongoing interpretations. Using annotation and memo functions, NVIVO 9 [52] maintained a permanent record of our work. Tracking individual responses in addition to the group account [53] assisted us in avoiding the risk of analyzing data from only vocally dominant members of the groups. Field notes or “descriptions of participants, impressions related to the discussion (and) observations related to group dynamics” [54] (page 85) maintained by both moderators during and immediately following the sessions further increased the dependability of our findings.

Practical issues such as organizing groups at a time and place to minimize disruption and avoiding power differential dynamics [55] were addressed. The groups were held when participants, who were normally separated by distance, were together in the same city for a required practicum experience. They were held at change of shift in lieu of a post conference. Knowing the power differential between students and teachers, moderators who did not have teaching responsibilities in the Post LPN to BN program were chosen to facilitate the focus groups. Instructors were not present during any of the discussions and had no involvement with the transcript data. Participants were recruited through a Letter of Invitation sent via email by a Research Assistant who was also not involved with the program. Four focus groups were held with 5 to 9 participants each. All the students who were invited chose to participate. We reasoned that this may have been because they were all from out of town and appreciated an opportunity to interact and share their views. Pseudonyms ensured participant confidentiality. Full ethical approval was granted by the university. The following two overarching themes emerged from analyzing the data. First, Post LPN to BN students need little, if any, further legitimation to affirm their identities as “nurse.” Second, practicum interactions with instructors and new clinical experiences are key socializing agents.

## 4. Results

### 4.1. Theme One: Post LPN to BN Students Need Little, If Any, Further Legitimation to Affirm Their Identities as “Nurse”

Without exception, participants in this project all commented on how they felt as though their identity as a “nurse” was well established before they entered the Post LPN to BN program. When invited to discuss memories of times when they felt affirmed in their identity as a “nurse,” several participants commented on skills they mastered in their practice as LPNs, for example:

*“When I gave my first injection—that was like—I'm a nurse!”*

*“I know it's the silliest thing, but doing the hospital corners for me was … very sentimental—I felt very nursy.”*

*“The gross stuff-wounds.”*

Participants discussed how others' expressions of trust in their knowledge also legitimized their identity as “nurse”:

*“Collaborating with the physicians. At my work, I would say I need this ordered, Dr … and he'll just say, okay, it's ordered. And then it seems like I make the decision and I just need his signature.”*

*“When the client would appreciate the care that you provide them, and also the family. They will speak with you and then thank you for whatever you did.”*

They talked about opportunities where demonstrating professional authority in their workplace further established their identity as “nurse”:

*“Working your first night shift … in your new role. The culture of night shift—it's different.”*

*“The first time I cared for a palliative patient and was there when they passed. Talking with the family. My first job that I had as an LPN, I was alone on the floor as the only official nurse for more than half of my shift. I had the full responsibility of all 60 residents in my care … that all happened as an LPN.”*

From the Post LPN to BN students' perspective, the notion that socialization into the role of “nurse” would occur for them at this point in their career was insulting:

*“I almost feel a little bit insulted to think that I would feel any less professional as an LPN than I do as an RN. I feel equally professional in both roles.”*

*“My buddy nurse actually asked me if I (implemented treatment) and I was like, I have done this before! It was a little like patronizing. I did not like being patronized in that sense. We are not newbies. We have been around.”*

*“We aren't newbie's. We do bring experience …. I'm still very proud of the work I do as an LPN. I already feel I do think like an RN. (It's) very frustrating and almost devalues the work that I've already put into the profession.”*

*“(In one course) the textbook was the exact same textbook that I used in my LPN course. Same cover, same everything. I found that so frustrating because I thought, I've read this textbook already.”*

In sum, Post LPN to BN students in our study expressed that they already viewed themselves as professional nurses. They were not “becoming nurses” by attending a university. One nurse offered this advice to those involved with educating this group of learners: “*It's extremely important when you are an adult learner to be treated as such. When you disregard our previous skill and knowledge, it's a blow to our ego, it's degrading*.”

### 4.2. Theme Two: Practicum Interactions with Instructors and New Clinical Experiences Are Key Socializing Agents

When participants in this study reflected on changes and growth in their professional identity, it was the practicum interactions with instructors and the opportunities for new experiences that stood out for them as particularly meaningful. Students consistently emphasized that they viewed the LPN and RN roles as similar during the focus group discussions. During the practical components of their program, it was especially important to receive legitimation from others that they were truly extending their existing “nurse” identity. Many students expressed that they did not feel different:

*“I think what's changing is how other people look at you more than how I feel, how other people treat you and how willing they are to give you responsibility versus how I ever felt.”*

*“I do not feel different myself. But people react to you differently. People are willing to give you more responsibility because you are going through the RN program.”*

*“I've noticed it's more external in how people treat you versus how you feel.”*

*“I do not feel different myself. But people react to you differently. People give you more responsibility.”*

Instructors expected students to demonstrate a capacity to seek out new and relevant information and frequently questioned them about their patients. This evaluation process was a familiar ritual to Post LPN students. When our participants felt that they responded well, they expressed a tentative willingness to risk identifying more with the RN role:

*“I find coming into this program, you need to justify everything that you're doing and explain the reason for it. It makes you think more about your reasons for doing something and whether you can justify them well enough to, you know, proceed to doing care. And I think maybe that's because you have somebody who's constantly challenging you to prepare. So if you can give a good answer, you know okay, I'm on the way. What can I do better? If you cannot answer the questions, then you're challenged to go and maybe research a little bit better so you do not feel, oh, I did not answer that question very well.”*

*“I think one thing that I find myself doing more just in this role is just researching because they put such an emphasis on that that I find myself looking everything up, so that's one thing that I've changed in my practice is I look things up more.”*

*“Well, as an LPN and an RN, it's our professional responsibility to have continuing competence and learning, so has taking the RN program taken me farther than I would have taken myself as an LPN? I do not know, because that was my responsibility to continue to learn … oh, I do not understand what this blood work means. Maybe I should look it up. I still should have and would have been doing that as an LPN. Going through this program has forced me to [research patient conditions] because we have homework every night to do, so maybe it's a little bit more forced learning but ….”*

*“Instructors have been really good about asking you questions and getting you to think or why is this being done, and why would you think that you would do this? And what would you do if this happened? (They) kind of encourage and draw out of me that way of looking at the whole picture.”*

Opportunities for new experiences supported participants' sense of gaining a more complex nursing identity. They identified the topic areas where they gained the most new knowledge as acute care, research, leadership, psychiatric mental health, and community nursing. Describing her opportunity to attend an Intensive Care Unit (ICU), one participant described how the prospect of interacting more with this patient group contributed to her growing identity as an RN: “*I went to ICU today, and like the whole day I was like Wow! This is awesome. I already work and feel confident in (acute care) I would have loved to (complete the practicum in ICU)*.”

Similarly, another participant described how she learned of a community resource which distributed milk to the needy and was able to refer her patient: “*After seeing what's out in the community …. I was never aware* (of these programs.) Another added: “*It's nice to see that nursing is the (profession) that does that! I get excited about it! There's so many things that we can do. (In hospital nursing) it's one on one. You help one person, but in community nursing, you're talking whole communities and populations, and like a long life span too right? They're going to teach their kids, and their children's children. That was interesting.”*

Participants consistently identified that the new experience of working alongside a Registered Nurse was also a powerful experience of legitimation: “*A good example today was (procedure). I did (procedure) so I got to sort of walk through that with my RN that I was working with. She was really, really open to sharing what she knew about it. (This opportunity was) definitely a new skill*.”

Where problems occurred in seeking new clinical experiences for Post LPN to BN students was the variance in their previous experience. It was during these discussions that the interactive nature of our focus groups was most apparent. Participants were interested in one another's perspective, but they did not agree on what actually constituted a “new” experience. Students who worked on acute care hospital units did not view some aspects of their university practicum as “*new*.” On the other hand, those who worked in long term care found the practicum “*very challenging*.” Problems also occurred when institutional policies for undergraduate nursing students did not take into account that Licensed Practical Nurses could also be members of this student group. Participants mentioned instances where “*my buddy nurse was an LPN with less experience than me*” and “*I cannot actually do some of the skills that I've been trained to do as an LPN in this practicum so it's kind of holding me back*.”

## 5. Discussion

The aforementioned two themes, developed from focus group discussions with Licensed Practical Nurses attending university to become Registered Nurses, begin to illustrate the experience of legitimation among this group of learners. Listening attentively as students described their experiences revealed useful ways to acknowledge their existing identity as nurses, to understand how important instructors' questions were to them and to conceptualize the notion of “new” experiences through their eyes.

Consistent with MacLellan et al.'s [56] research with dietetic students and Klossner's [5] research with student athletic trainers, our project also revealed that professional socialization begins when instructors and patients accept students in their new professional role. This acceptance and acknowledgement by others generates confidence and a willingness to risk behaviours expected of those in the new role. Similarly, our project echoes Spoelstra and Robbins [28] research with Registered Nurses transitioning to an advanced practice role. Like our participants, the practising nurses in Spoelstra et al.'s study identified that implementing direct patient care was an essential component in their successful role transition.

However, the experiences of legitimation that Post LPN to BN students face are unique. Traditionally, university programs did not offer bridging programs to vocationally educated nurses. In turn, questions about the legitimacy of their new program may be raised. Scales measuring values new students acquire as part of their socialization into the role of nurse, such as Weis and Schank's [57] Nursing Professional Values Scale-Revised NPVS-R or Shinyashiki et al.'s [58] professional socialization questionnaire, are not fitting for this group of nursing students. Licensed Practical Nurses begin their program already well socialized into the identity of “nurse.” Participants in the present study felt insulted by the notion of “becoming” a nurse. Given the similarities between the LPN and RN role in their workplaces, they did not always feel that they were doing anything “different” in their practicum.

Affirmation from others that their professional identity was extending and changing was especially important to this unique group of learners. The authenticating experiences of completing courses and examinations in cohort groups that Haas and Shaffir [4] considered foundational to professional socialization were not available to these students. They completed prepracticum courses and examinations alone and online. Their employment experiences as LPNs did not usually support acting independently. On their practicum units, their name badges did not effectively communicate what their role was. In some instances, they were prevented from implementing nursing care that was part of their everyday practice. In essence, the typical legitimation agents that historically supported healthcare learners towards new professional identities are not fully available to Post LPN BN students. Therefore, both the time they spent with instructors and “new” clinical experiences were especially important.

Limitations of the study included recruiting a small homogenous sample of learners from only one program. As we were not previously acquainted with participants, group dynamics such as dominance by one or two members, power differentials, or established patterns of communication may have influenced the conversations. Despite our moderators' attention to group process, some participants may have simply agreed with others, not expressed their views fully, or commented only superficially.

## 6. Conclusion

Given these findings, implications for instructing Post LPN to BN students include honoring the feelings of legitimacy they have already developed as practising professional nurses. Educators must ensure that opportunities are available for these learners to meet with their instructors regularly and to engage them in learning topics and experiences that, in their view, are “new.”

Clearly, structured evaluation times are a priority. Students expect and need the formal acknowledgement that they are progressing or not progressing as expected. Although students may not “feel different” themselves, acknowledgement from others that they are developing a new nursing identity can be impactful. Further, the importance of encouraging students to identify individual learning goals should not be underestimated. Traditional undergraduate placements cannot be expected to accommodate all the needs of this diverse group of adult learners. It is critical for educators to recognize that “new” experiences are likely to be different for each student.

In conclusion, this paper presented findings from a descriptive study that explored Post LPN to BN students' experiences with professional socialization. The research investigated socializing agents that impacted students' feelings of legitimacy as they developed new identities as Registered Nurses. In contrast to other studies, this project extends our understanding of healthcare learners' professionalization by including the voices of Licensed Practical Nurses who attended university. Knowing the value that this group of adult learners place on instructor evaluation and “new” clinical experiences, implications for educators include ensuring that one-to-one time with their teachers is available and designing practicum experiences that build on their established identities as professional nurses.
